# Supplementary material for: Right-to-Left Shunt in Divers with Neurological Decompression Sickness: A Systematic Review and Meta-Analysis
Source: Healthcare (Basel). 2023 May 12;11(10):1407. doi: 10.3390/healthcare11101407 (PMC10217987; doi:10.3390/healthcare11101407)
Supplement: Supplementary file 1 [file healthcare-11-01407-s001.zip › healthcare-2246030-supplementary.pdf]

Supplementary Table 1. Risk of bias assessment according to Quality in Prognostic Studies (QUIPS) tool

Study	Study participation	Study attrition	PF measurement	Outcome measurement	Study confounding	Statistical analysis and reporting
Cantais 2003	Low	Low	Moderate	Moderate	Moderate	Low
Cartoni 2004	Moderate	Low	Low	Moderate	Moderate	Low
Gempp 2008	Moderate	Low	Moderate	Low	Moderate	Low
Gempp 2017	Low	Low	Moderate	Low	High	Low
Germonpre 1998	Low	Low	Low	Low	Moderate	Low
Koch 2008	Low	Low	Moderate	Low	Moderate	High
Liou 2015	Moderate	Low	Low	Low	High	Low
Torti 2004	Low	Low	Low	Low	Moderate	Low
Wilmshurst 1989	Moderate	Low	Low	Moderate	Moderate	Moderate
Wilmshurst 2000	Moderate	Low	Low	Moderate	Moderate	Moderate
Germonpre 2021	Moderate	Low	Moderate	Low	Moderate	Low
Balestra 2021	Low	Low	Low	Low	Moderate	Low
Gempp 2010	Low	Low	Moderate	Low	Moderate	Low
Gerriets 2003	Moderate	Low	Moderate	Low	Moderate	Low
Knauth 1997	Moderate	Low	Moderate	Low	Moderate	Low
Koch 2004	Moderate	Low	Low	Low	Moderate	Low

PF: prognostic factor
